# Regulation of HPV transcription

**DOI:** 10.6061/clinics/2018/e486s

**Published:** 2018-09-26

**Authors:** Aline Lopes Ribeiro, Amanda Schiersner Caodaglio, Laura Sichero

**Affiliations:** Centro de Pesquisa Translacional em Oncologia, Instituto do Cancer do Estado de Sao Paulo (ICESP), Hospital das Clinicas HCFMUSP, Faculdade de Medicina, Universidade de Sao Paulo, Sao Paulo, SP, BR

**Keywords:** Human papillomavirus (HPV), Transcription, Long Control Region, Differentiation

## Abstract

Human papillomavirus infection is associated with the development of malignant and benign neoplasms. Approximately 40 viral types can infect the anogenital mucosa and are categorized into high- and low-risk oncogenic human papillomavirus, depending on their association with the development of cervical carcinoma. High-risk human papillomavirus 16 and 18 are detected in 55% and 15% of all invasive cervical squamous cell carcinomas worldwide, respectively. Low-risk human papillomavirus 6 and 11 are responsible for 90% of genital warts and are also associated with the development of recurrent respiratory papillomatosis. Human papillomavirus preferentially infects mitotic active cells of the basal layer from both mucosal and cutaneous epithelium through microabrasions. The viral life cycle synchronizes with the epithelial differentiation program, which may be due, in part, to the binding of differentially expressed cellular transcription factors to the long control region throughout the various epithelial layers. This review aimed to summarize the current knowledge regarding the mechanisms by which viral gene expression is regulated and the influence of human papillomavirus heterogeneity upon this phenomenon. A better understanding of the regulatory mechanisms may elucidate the particularities of human papillomavirus-associated pathogenesis and may provide new tools for antiviral therapy.

## INTRODUCTION

Human papillomavirus (HPV) infection is strongly associated with the development of several malignant and benign neoplasms. Over 200 different HPV genotypes have been isolated, thoroughly sequenced and numbered in order of their discovery, among which approximately 40 types can infect the anogenital mucosa 1. These types are also categorized into high- and low-risk oncogenic HPVs based on their association with the development of cervical carcinoma. HPVs 16, 18, 31, 33, 35, 39, 45, 51, 52, 56, 58, 59 and 66 are classified by the International Agency for Research on Cancer (IARC) as carcinogenic in humans (type I carcinogens), while HPVs 6, 11, 40, 42, 43, 44 and 54 are grouped as low-risk oncogenic viruses 2.

The association between HPV infection and the development of cervical cancer was first reported in the early 1970s 3. Although most infections are asymptomatic and spontaneously eliminated, persistent infections by high-risk HPVs are responsible for the development of most, if not all, cervical cancers worldwide 4. HPVs 16 and 18 account for approximately 55% and 15% of all invasive cervical squamous cell carcinoma (SCC) worldwide, respectively 5. Furthermore, approximately 85% of anal canal tumors, 50% of vulvar and penile tumors and 70% of vaginal tumors can be attributed to HPV infection, in addition to 10% to 90% of oropharynx cancers. It is noteworthy that HPV-16 is detected in almost all cancers of these extracervical anatomical sites that are associated with HPV infection 6,7. On the other hand, low-risk HPVs 6 and 11 are responsible for over 90% of genital warts (GWs) in both genders and are also associated with the development of recurrent respiratory papillomatosis (RRP), which is characterized by the repeated formation of benign papillomas in the upper respiratory tract 8,9. Nevertheless, although classified as low-risk, HPV-6 has been identified in several malignancies, including carcinomas of the vagina 10, vulva 11,12, penis 13, tongue 14, cervix 15,16, and tonsils 17.

HPVs are nonenveloped viruses with a circular, double-stranded genome that comprises approximately 8000 base pairs (bp) 18. The viral genome is physically divided into 3 regions: the early (E) and late (L) regions and the long control region (LCR). E1 and E2 proteins play a role in regulating viral DNA transcription and replication 19, whereas E4, E5, E6, and E7 proteins are involved in cell cycle deregulation, immune evasion and recruitment of replication host factors 20. L1 and L2 late proteins comprise the major and secondary capsid proteins, respectively.

HPVs infect the stratified squamous epithelia, both mucosal and cutaneous, and preferentially target mitotically active cells of the basal layer through microtraumas. In basal epithelia cells, HPVs are established as episomes within the nuclei, and expression of early proteins that are necessary for DNA replication occurs 20. As basal cells undergo differentiation, the expression pattern of HPVs is modified. Finally, in more differentiated cells, late gene expression occurs, and new virions are released 21. The switch between HPV promoters activation in the cells of supra basal layers suggests that synchronization of the viral life cycle to the epithelial differentiation program is due to, at least in part, the binding of a repertoire of differentially expressed cellular transcription factors (TFs) to the LCR throughout the various layers of the epithelium.

This review summarizes the current knowledge regarding the mechanisms of viral gene expression regulation at the transcription level and the influence of HPV heterogeneity upon this phenomenon. Advances in the knowledge of the regulatory networks may shed light on the particularities of HPV-associated pathogenesis that are relevant for disease establishment and may provide new tools for antiviral therapy.

### The long control region

The LCR is a noncoding region between the *L1* and *E6* genes that comprises approximately 10% of the viral genome and is physically divided into three distinct segments: the 5′ segment, the central segment and the 3′ segment.

Most mucosal HPVs present four E2 binding sites (E2bs) distributed along all the three LCR regions. The 5′ segment contains the first E2bs, in addition to the transcription termination and polyadenylation sites for late transcripts. The central segment of the LCR is flanked by two E2bs and has been shown to function as an epithelial-specific transcriptional enhancer 22-24. This segment encloses several motifs that are important either to stimulate or to suppress viral transcriptional activity. These motifs include binding sites to AP1, NF1, TEF1, OCT1, YY1, BRN-3a, NF-IL6, KRF-1, NF-kB, FOXA1, and GATA3, aming several others 25-28. Thus, the activation/repression of HPV early promoters involves synergism between these proteins, which vary in affinity for the different cis-elements within the LCR of the different HPV types and variants. Finally, the 3′ segment of the LCR contains two E2bs in addition to an E1 binding site (E1bs) overlapping the origin of replication.

**Figure 1:**
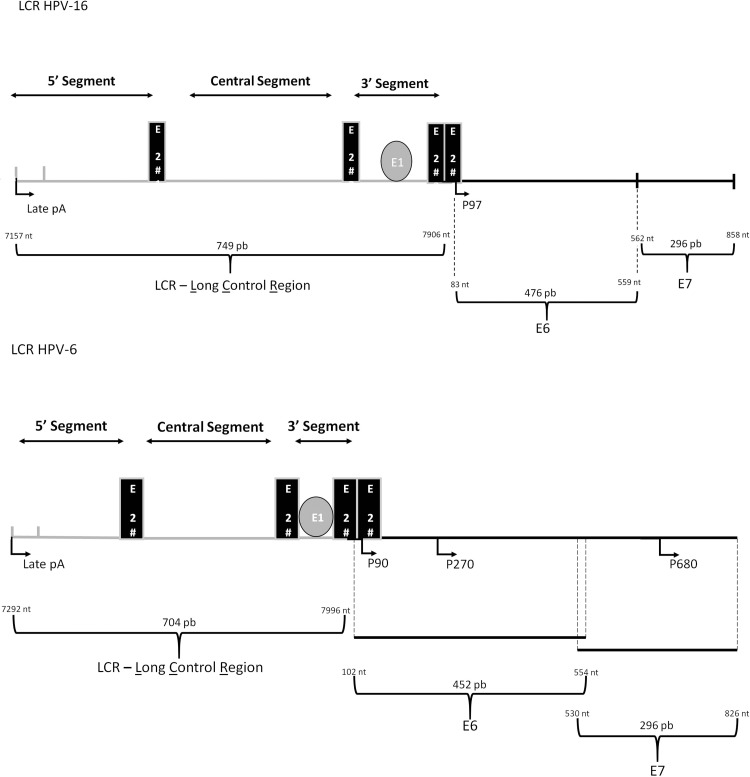
Schematic representation of the LCR and E6 and E7 genes from high- and low-risk HPVs, represented by HPV-16 and HPV-6, respectively. Four E2 binding sites (E2bs) are conserved among mucosal HPVs, with E2bs#1 and E2bs#2 dividing the LCR in three distinct segments: the 5' segment, the central segment and the 3' segment. The 5' segment contains the late transcription termination signal, denominated ‘late pA', the central segment functions as an epithelial-specific enhancer, and the 3' segment encloses the ori region. Early promoters are also indicated: HPV-16 (P97) and HPV-6 (P90, P270, P680).

P97 and P105 are the main early promoters of HPVs 16 and 18, respectively, and are also the most well studied. For both high-risk HPVs, transcripts initiated in E6 are differentially edited, codifying all early genes 29 (Figure 1). Additionally, for both viral types a part of the E6 mRNA is spliced out for efficient translation of E7 30.

In contrast, in low-risk HPVs 6 and 11, two early promoters have been identified, P90 and P270, that enable the individual regulation of *E6* and *E7* genes, respectively, although the regulation of both promoters is controlled by cis-elements within the LCR 30-35. It is noteworthy the presence of a third early promoter in HPVs 6 and 11, P680, that encodes the fusion protein E1^∧^E4 36. It has been reported that P90, P270 and P680 are differentially regulated, indicating that the independent regulation of early proteins is important to the viral life cycle 32,34,37-39. For example, the binding of E2 to the most distal E2bs stimulates P270, whereas E2 binding to both proximal sites inhibits the P90 E6 promoter 38. Furthermore, the P680 promoter was shown to be used in a differentiation-specific manner both *in vitro* and *in vivo*. Interestingly, whereas E7 mRNA was the most abundant transcript in a cervical carcinoma sample, in a benign genital wart, E1^∧^E4 transcripts were the most prevalent 15.

### Transcriptional regulation of HPVs

Transcription is the first step of gene expression control, followed by regulation of mRNA processing and other posttranscriptional events. Transcriptional regulation not only restricts the expression of early and late genes to when they are needed but also limits the range of cell types that HPVs are able to infect, avoids the premature expression of immunogenic viral proteins, and modulates virus biology in response to hormones and growth factors 40-42. HPV transcription is mostly controlled by the E2 viral protein in addition to host cells TFs that bind to specific sequences within the LCR which vary substantially among different viral types and variants 43. HPV gene expression control also involves epigenetic changes, such as nucleosome remodeling and DNA methylation 44.

Approximately 90 bp downstream of the E1 binding site is where the transcription start site is located. A segment of approximately 45 bp within these 90 bp contains a SP1 binding site overlapping the E2bs#3 and a TATA box overlapping E2bs#4. The overlap of both E2bs with these cis-elements occurs in such a way that occupancy by E2 proteins displaces TFIID and the SP1 factor 45-49. In turn, the binding of SP1 and TFIID to the LCR sterically inhibits E2 binding, activating early promoters 46,49. In all mucosal HPVs, the spacing of the SP1, TATA, and E2 binding sites at E6/E7 promoters are conserved 28.

E2 is the most important viral transcriptional regulator in HPV early expression and therefore has a high impact on E6 and E7 protein levels 25,50. In preneoplastic and malignant neoplastic lesions, the viral DNA often integrates into the cell genome 51, and integration seems to occur mostly at random 52. However, viral integration usually results in interruption of the E2 gene, abolishing E2-negative transcriptional regulation and resulting in constitutive expression of E6 and E7 proteins 25.

In addition to binding directly to its specific sequence, E2 also influences viral transcription by recruiting cellular factors to the viral genome. One of the best-characterized host interactors of E2 is the bromodomain-containing protein 4 (Brd4), a transcription cofactor and chromatin regulator 53. Both E2-mediated activation and repression of the early promoter require interaction with Brd4 54,55. A recent study revealed that a phosphorylated region of Brd4 interacts selectively with high-risk E2 proteins. The study showed that the blockage of phospho-Brd4 activity alleviates E2-mediated inhibition of HPV-18 promoter activity 56. Another study demonstrated that Brd4 is capable of directly activating HPV-18 transcription during early stages of infection in an E2-independent manner, indicating that Brd4 plays a central role in the dynamics of viral expression 57.

Another important virally expressed transcriptional regulator is the E8^∧^E2 protein, which is a product of splicing 58. It was demonstrated that E8^∧^E2 proteins in HPVs 16, 18, and 31 are potent repressors of viral transcription, and the conserved E8 component of E8^∧^E2 is capable of recruiting cellular corepressors to inhibit transcription of the viral major early promoter in such a way that is more efficient than the recruitment by the E2 component 59-61. Host cell NCoR/SMRT complexes have been reported to act as corepressors of E8^∧^E2 proteins in HPVs 1, 8, 16 and 31 62. This complex encloses GPS2, HDAC3, NCoR, SMRT and TBl1 and TBLR1 proteins and is typically involved in transcriptional repression of cellular genes.

Concerning cellular TFs that bind directly to the LCR, there is a large list of well-characterized regulators (including YY1, AP1, NF1, OCT1, SP1, and CDP), many of which conservatively bind to the central segment of the LCR of several HPV types that infect the mucosa. These regulators have been demonstrated to be relevant not only to HPV epitheliotropism but also to host cell differentiation-dependent regulation of viral infection 25. It was recently reported that the cellular CCCTC-binding factor (CTCF) has a conserved binding site within the E2 open reading frame of high-risk HPVs, in addition to a cluster of cis-elements in the late gene region of the genomes of HPVs 16, 18, 31, 11, and 6. CTCF is recruited during epithelial differentiation to regulate both the transcription of E6 and E7 and transcript processing 63.

García-Vallvé and col. (2006) performed an in silico analysis to search for putative cis-elements within the LCR of 61 papillomavirus (PVs) types infecting 20 different hosts 43. Overall this study indicated that the number and nature of TF binding sites within the LCR can be much broader than described to date, some of which are predicted to be present in most PVs, whereas others cis-elements seems to be restricted to specific PVs. Curiously, in this study, YY1 putative binding sites in the LCR of HPVs 18 and 6 were not found, although the impact of this TF upon the transcriptional activity of both viral types was already demonstrated 64,65. It is noteworthy, however, that the identification of a putative cis-element alone does not implicate that this nucleotide sequence will always be bound by its cognate TF. For instance, many cis-elements within the various LCRs are juxtaposed or even overlapped with others. We must also take into consideration the existence of degenerate and/or low affinity sites that could increase the number of probable binding sites for a given protein. Furthermore, some TF binding sites are very similar, such as the recognition sequences for TEF-1, TEF-2 and YY1, and these sites may be differently occupied depending on the availability of each of these proteins 28.

Additionally, the HPV LCR embraces hormone-responsive elements, including glucocorticoid response element (GREs) and progesterone responsive elements (PREs). Complexes formed by steroid hormones and receptor proteins interact with these specific regulatory sequences and either upregulate or downregulate the transcription of viral early genes 66,67. The administration of exogenous estrogen induces HPV-18 LCR activity in both squamous and glandular cells of the cervix and vagina, leading to an increased E6/E7 expression and to a higher susceptibility of neoplastic transformation 68. Likewise, a study regarding smoking-related effects upon the LCR activity revealed that tobacco smoke was able to activate the HPV-16 P97 promoter in a dose-dependent manner in tumor lung cells. However, in nontumor lung cells, the same effect depended on the ectopic expression of HPV-16 E6 and E7 oncogenes 69.

Chromatin remodeling also plays a pivotal role in regulating HPV transcription. The HPV-16 genome contains nucleosomes in specific positions, one at the center of the viral enhancer and another overlapping the replication origin and the E6 promoter 44. Both nucleosomes repress the activity of the early promoter, which is released by the addition of SP1 and AP1 proteins 70. The influence of nucleosomes upon viral early and late transcription events was also reported in HPV-31: nucleosomes at both promoter regions were activated through histone modifications during differentiation 71.

In addition to the remodeling of nucleosomes, DNA methylation also participates in the epigenetic regulation of HPV gene expression. Host cell methyltransferases methylate HPV DNA, and viral DNA methylation profiles have been related to important features of the viral life cycle 72. Among these features, several studies have focused on the analysis of E2bs methylation, and taken together, the data reveal that differential methylation of the different E2bs has an impact on the activation of viral E6 and E7 expression in cervical lesions 73-75. Additionally, analysis of the methylation status of CpG dinucleotides within TF binding sites in the LCR of HPV-16 indicated that these epigenetic alterations are linked to squamous epithelial differentiation 76.

While most studies regarding HPV transcriptional control rely on early promoter activity, little is known about the mechanisms regulating late promoters. The levels of a variety of cellular TFs, including c-MYB, NF1, PAX5, NFAT, STAT5, C/EBPβ, among others, increase upon epithelial differentiation; however, the functional relevance and how these various factors contribute to transcription regulation remains unclear 77,78. Recent findings indicate that transcript elongation is also a critical step for viral late gene upregulation. It was demonstrated that cyclin dependent kinase 9 (CDK9), CDK8 and Brd4 are recruited to activate the HPV-16 late promoter under differentiation conditions and to assist Pol II complex activity until transcription is accomplished 79. The viral oncoprotein E7 might also activate the HPV-16 late promoter, indicating that the virus, *per se*, also plays an active role in regulating its own expression 80.

The study of HPV regulatory elements progressed rapidly for approximately 15 years, beginning in the early 1980s, but interest declined dramatically, which was possibly associated with limitations of the available methodologies. However, the understanding of HPV transcriptional regulation is far from achieving maturity. In the last decade the development of array technologies made possible the identification of several additional TFs that impact HPV transcriptional activity. Competition assays using an array comprising 345 consensus binding sites for different TFs identified factors that specifically bind to the HPV-16 LCR in differentiated and undifferentiated cells 77. It is noteworthy that the methodology used was able to confirm several TFs already described to influence the expression of HPVs, although most of the associations reported were still unpublished.

In our laboratory a powerful approach was used to analyze the effect of 704 TFs upon the transcriptional activity of HPVs 16 and 18 using a large-scale transfection assay 26. Twenty-eight TFs that stimulated and 36 that repressed the LCR were identified, most of which had not yet been described. Although many of the TFs appear to act by indirect mechanisms, binding sites on the viral LCR for a subset of these modulators were identified. Among these, FOXA1 had the greatest effect upon HPV-16 and HPV-18 transcriptional activities, and this was a direct effect, as we were able to show *in vivo* binding of FOXA1 to the LCR in both viral types. Moreover, the differential expression patterns of FOXA1 indicate that this protein may be important to HPV-associated carcinogenesis: we observed that FOXA1 is expressed at higher levels in immortalized and HPV-transformed cell lines than it is in normal cells 26, and FOXA1 was further shown to be strongly expressed in basal epithelial cells, preinvasive lesions, and cervical and head and neck carcinomas 81. Sex determining region Y (SRY)-box 2 (SOX2) has also been recently proposed as a transcriptional repressor of the HPV-16 LCR; SOX2 binds to three putative binding sites identified in the enhancer sequence of the LCR through direct interactions and leads to the inhibition of E6 and E7 expression 82.

### Impact of HPV nucleotide heterogeneity upon LCR transcriptional activity

HPV taxonomy is based on the variability of the *L1* gene sequence, and viral genomes are classified as new types when these have less than 90% of identity with any other type. Additionally, genome heterogeneity of 1.0-10.0% and 0.5%-1.0% define HPV variant lineages and sublineages, respectively 83. HPV DNA variability studies have been used as an important tool for the analysis of viral evolution. Additionally, it has been observed that specific HPV variants are differentially associated with disease outcome. Since HPV variants show approximately 5% of divergence within the LCR, the impact of HPV nucleotide heterogeneity upon transcriptional activity has been analyzed since nucleotide divergence could influence TF affinity for their recognition sites, thus influencing E6/E7 levels and consequently the carcinogenesis induced by these viruses. In fact, we reported that the HPV-18 P105 promoter is 12 times more transcriptionally active than the HPV-16 P97 promoter 84. These results corroborate previous observations showing that although HPV-18 is 10 times more efficient than HPV-16 in the transformation of human keratinocytes, E6/E7 proteins of both viral types immortalizes these cells with similar efficiency when expressed from a heterologous promoter 85.

Since the early 90s it is know that nucleotide alterations within the LCR among variants of HPV-16 overlap cis-elements 86. Next, it was reported that whereas variants from the A1-A3 sublineages have similar transcriptional activity, variants from the D sublineage attain higher activity, which may support augmented E6 and E7 levels and finally confer enhanced oncogenic potential to specific variants 87. These observations are in line with epidemiological data that indicate that HPV-16 variants from the D lineage are associated with an increased risk of HPV persistence and cervical disease development 88-95. Differences in transcriptional activity were attributed to the E6-proximal end of the LCR, even though it was not possible to implicate the observed differences to a single nucleotide alteration 96. It is also important to note that TF recognition sequences could be created or abolished between HPV variants due to nucleotide changes in the LCR. The expression of viral E2 and E1 replicating proteins has also been influenced by the heterogeneity in the LCR sequence inherent of HPV-16 variants, which has affected the viral replication efficiency as well 97.

BRN3, a long-known cellular transcription factor, binds directly to a specific motif of the HPV LCR, strongly stimulating its activity 98,99. BRN3 and nicotine from smoking were shown to have a synergistic effect upon the LCR in a variant-specific manner 100. The BRN3/nicotine response increases the transcription HPV-16 *E6*/*E7* genes and was shown to be related to higher grades of cervical intraepithelial neoplasia and cancer.

**Figure 2:**
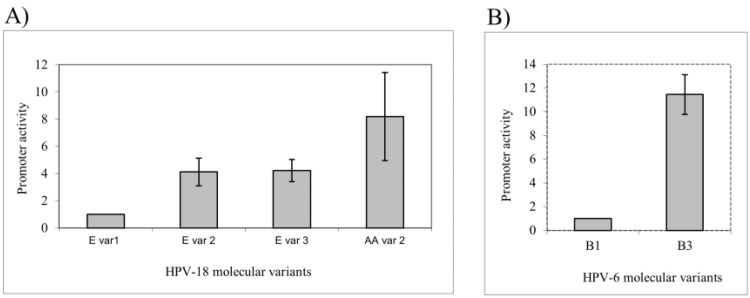
Differential promoter activity among different HPV variants. (A) Variants from the HPV-18 Asian-Amerindian branch presented a higher early promoter activity than variants of the European branch. (B) For HPV-6, a variant of the B1 sublineage was ten times more transcriptionally active than was a variant from the B3 sublineage. Adapted from Sichero et al., 2005 84 and Measso do Bonfim et al., 2015 105.

Although it was observed that the LCR sequence of HPVs 18 and 58 is more conserved than the LCR sequence of HPV-16, there are significant differences in the early promoter activity among molecular variants of both viral types 84,101. For instance, we observed that Asian-Amerindian variants achieved higher transcriptional activity than variants from the European branch 84 (Figure 2A).

Genome variability of low-risk HPVs 6 and 11 has also been evaluated, and molecular variants of both viral types were phylogenetic grouped 102-104. It was reported that a variant from the HPV-6 B1 sublineage is ten times more transcriptionally active than a B3 variant is 105 (Figure 2B). Since a significant association between variants of the B1 sublineage and the development of GWs was recently described, it is possible to hypothesize that the increased transcriptional efficiency could impact the increased expression of viral E1 and E2, which can confer B1 variants to have an increased replication potential 106. Moreover, the results support a crucial role of the ELF1 protein in the lower transcriptional activity observed for the B3 variant 105. A correlation between the transcriptional activity of specific HPV-11 variants and the clinical aggressiveness of RRP has also been reported 107. Additionally, it was reported that duplication in the early viral promoter sequence of HPV-11 was associated with a higher degree of disease severity 108.

A broader comprehension of HPV mRNA expression and its regulation could lead to the development of novel diagnostic approaches and reveal strategic cellular targets for the development of innovative antiviral therapies 25. Even though inhibition of transcriptional regulatory factors could be harmful to normal cells once it is required for host cell homeostasis, these proteins could be inhibited using compounds targeting specific LCR sequences, thus affecting viral life cycle. Therefore, a molecule that could specifically bind to cis-responsive elements indispensable for *E6* and *E7* transcription of different viral types and variants could interfere with the transformation process that is dependent on the constitutive expression of both oncoproteins.

A recent approach based on TF research relied on the identification of molecular signatures associated with HPV positivity and the prognosis of head and neck cancers outcome. In this context, differential expression of key transcription factors, such as AP1, activator of transcription 3 (STAT3) and NF-κB, was reported between HPV-positive and HPV-negative cancers 109-111. More specifically, in HPV-positive squamous cell carcinomas of the oral cavity, higher levels of AP1 and NF-κB in addition to a lack of STAT3 was observed, and this pattern was suggested to be useful for discriminating tumors with a better prognosis 110. Therefore, the study of TF expression patterns may also provide useful prognostic biomarkers as well as novel insights to the molecular aspects of HPV-driven carcinogenesis.

## AUTHOR CONTRIBUTIONS

Ribeiro Al, Caodaglio AS, and Sichero L critically discussed and wrote the manuscript.
